# Intelligent Rolling Bearing Fault Diagnosis Method Using Symmetrized Dot Pattern Images and CBAM-DRN

**DOI:** 10.3390/s22249954

**Published:** 2022-12-17

**Authors:** Wei Cui, Guoying Meng, Tingxi Gou, Aiming Wang, Rui Xiao, Xinge Zhang

**Affiliations:** School of Mechanical Electronic & Information Engineering, China University of Mining & Technology, Beijing 100083, China

**Keywords:** rolling bearings, intelligent fault diagnosis, symmetrized dot pattern (SDP), deep residual network (DRN), attention mechanisms

## Abstract

Rolling bearings are a vital component of mechanical equipment. It is crucial to implement rolling bearing fault diagnosis research to guarantee the stability of the long-term action of mechanical equipment. Conversion of rolling bearing vibration signals into images for fault diagnosis research has been a practical diagnostic approach. The current paper presents a rolling bearing fault diagnosis method using symmetrized dot pattern (SDP) images and a deep residual network with convolutional block attention module (CBAM-DRN). The rolling bearing vibration signal is first visualized and transformed into an SDP image with distinct fault characteristics. Then, CBAM-DRN is utilized to derive characteristics directly and detect faults from the input SDP images. In order to prevent conventional time-frequency images from being limited by their inherent flaws and avoid missing the fault features, the SDP technique is employed to convert vibration signals into images for visualization. DRN enables adequate extraction of rolling bearing fault characteristics and prevents training difficulties and gradient vanishing in deep level networks. CBAM assists the diagnostic model in concentrating on the image’s more distinctive parts and preventing the interference of non-featured parts. Finally, the method’s validity was tested with a composite fault dataset of motor bearings containing multiple loads and fault diameters. The experimental results reflect that the presented approach can attain a diagnostic precision of over 99% and good stability and generalization.

## 1. Introduction

Vibrational faults are common in large machinery and equipment, affecting their normal operation and increasing maintenance costs. In severe cases, it can even cause mechanical damage and substantial economic losses or cause casualties. Rolling bearings are an essential mechanical equipment component and one of the most frequently faulty components. Faults in rolling bearings can deviate the entire mechanical system from normal working conditions, leading to downtime, production stoppage, and equipment damage [1]. In order to detect faults in time, different signals are collected using sensors to analyze and diagnose the operating conditions of rolling bearings. The development of rolling bearing (RB) fault diagnosis (FD) research significantly affects the long-term stable efficiency of mechanical equipment [2,3].

The rolling bearing fault diagnosis (RBFD) research is based on feature extraction and pattern recognition. The temperature signal, electrical signal, acoustic emission signal, vibration signal, and other signals can be employed for the feature extraction stage [4]. Due to its ability to respond rapidly to variations in the state of mechanical equipment, vibration signal analysis has the advantages of high accuracy and easy acquisition. Thus, as one of the primary analysis methods, it has been employed to derive bearing fault characteristics. With the rapid progress in signal processing technology, many methods such as time, frequency, and time-frequency domain analyses have been extensively utilized in the rolling bearing vibration signals’ processing and analysis [5,6,7,8]. In the pattern recognition stage, methods such as BP neural networks [9], fuzzy neural networks [10], and support vector machines (SVM) [11] have also been extensively utilized in the RBFD. Nevertheless, the mentioned models belong to a shallow network structure, resulting in the lack of the model’s fault diagnosis ability and generalization [12].

By constructing a multi-layer nonlinear network structure, deep learning methods can effectively simulate and approximate any complex function. Since fault characteristics can be derived adaptively from the primary data, it has been extensively utilized in several areas in recent years. The convolutional neural network (CNN), one of the representative branches of deep learning, has been utilized by many researchers in the RBFD [13]. Taking the primary multi-channel signals as the input, She et al. presented a multi-channel deep CNN using the exponential decay learning rate for verifying the RBs’ health status [14]. Janssens et al. applied the raw frequency domain data as the input to a CNN model to achieve the RBFD in four kinds of rotating machinery operating conditions [15].

According to the characteristics of CNN suitable for image recognition, the vibration signals of rolling bearings can be transformed into feature images for the RBFD. This approach transforms the RBFD problem into a multi-classification problem of rolling bearing vibration signal images efficiently and accurately [16]. The time-frequency analysis approaches like Short Time Fourier Transform (STFT), Wavelet Transform (WT), and Wigner-Vill Distribution (WVD) are generally employed to transform the vibration signal into feature images to apply the input into the CNN model [17,18,19,20,21]. However, due to linear time-frequency analyses approaches such as STFT and WT with unstable time-frequency resolution, the non-linear time-frequency analysis methods represented using WVD are susceptible to inherent cross-term interference [16]. Therefore, some vibration signal visualization methods, such as the axial trajectory method [22] and Symmetrized Dot Pattern (SDP) analysis [23], were applied to vibration signal feature extraction. SDP analysis provides a simple and intuitive way of converting the original vibration signal into an image comprising mirrored symmetry points in polar coordinates. In order to better reflect the vibration signal’s changing features, it is directly converted into SDP images in polar coordinates. Sun et al. adopted the SDP analysis to transform the vibration signals and classify different vibration signals of RBs by combining the improved Manhattan distance and the improved Chebyshev distance, respectively [24,25]. Zhu et al. transformed time series data from multiple sensors into SDP images and employed CNN to identify rotor vibration faults with different vibration states [26]. Gu et al. utilized the SDP to visualize the angular domain denoised reconstructed signals and DCNN to classify the formed SDP images to achieve RBFD under various working conditions [27]. However, extracting features from faulty signals is insufficient because the employed CNN model only contains a limited number of convolutional and pooling layers.

With the increasing volume of bearing condition monitoring data, researchers have employed the more powerful feature extraction capabilities of Deep Residual Networks (DRN) to diagnose faults of bearings in complex operating conditions to process more information. An approach using time-frequency analysis and the deep residual network was presented for the FD of planetary gearboxes [28]. Ref. [29] trained a deep ResNet for RBFD via the raw vibration signals as the model input. The results indicated its superiority to traditional CNN models. In [30], a multi-scale kernel-based residual convolutional network was presented for motor fault diagnosis considering the machine’s non-smooth conditions. The results indicated the superiority of the method over traditional approaches. Nevertheless, the RBFD using DRN as a diagnostic model has not been widely studied, while the diagnostic performance could still be improved using attention mechanisms, fine-tuned network architectures, and other methods.

According to the mentioned analysis, the current paper proposes an RBFD approach using the SDP image and deep residual network with convolutional block attention module (CBAM-DRN). Firstly, the bearing vibration signal is converted into an intuitive SDP image with apparent features. Then, the diagnostic model of CBAM-DRN is established to extract features and classify faults using the SDP image. The DRN model avoids the performance degradation of normal networks due to the high number of layers. The added CBAM attention mechanism allows the model to focus on regions with more significant fault characteristics, reducing the interference of redundant information. Finally, motor-bearing datasets containing different loads and fault diameters verify the proposed method through experiments. The presented approach has better diagnostic accuracy and generalization performance than the traditional models. The advantages of the proposed bearing fault diagnosis approach as follows.

(1) Transforming bearing fault diagnosis research to the classification of bearing fault images and proposing an intelligent fault diagnosis approach by combining SDP images with the CBAM-DRN method.
(2) The optimal SDP parameters are selected to visualize the bearing vibration signal and convert it to the SDP images with obvious fault characteristics.
(3) Combining the advantages of attention mechanism and deep residual network, the approach can automatically and comprehensively extract the deep fault features in the image and improve the diagnostic accuracy.

The rest of the paper is divided into the following sections. Section 2 describes the basic theory of the method used in this paper. Section 3 describes the proposed method for bearing fault diagnosis using SDP images and CBAM-DRN. Section 4 gives the data set and model parameter settings used, validates the effectiveness and superiority of the proposed approach, and presents the experimental results and comparative analysis. Finally, conclusions are given in Section 5.

## 2. Theory

This section introduces the fundamental theory of the SDP, DRN and CBAM methods used in this paper.

### 2.1. SDP Method

Using the SDP method to generate images, the variation in the sampled signals’ amplitude and frequency can be verified via differences in the images. Since it does not need to undergo time-frequency analysis and only operates on time-domain signals, it has the advantages of simplicity, convenience, and fast calculation.

**Figure 1:**
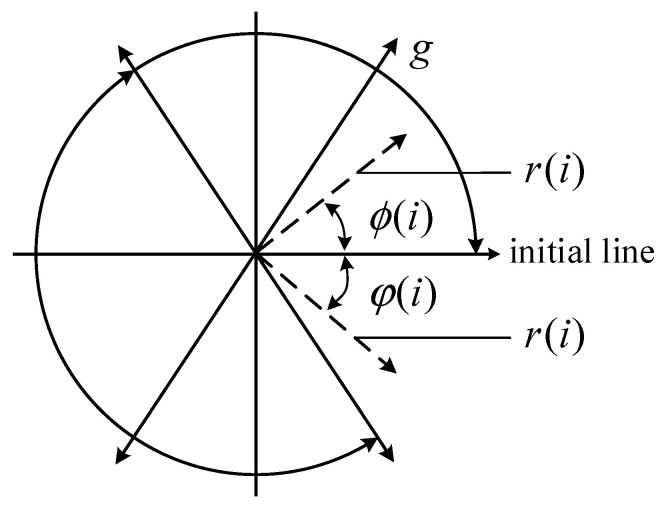
Schematic diagram of the SDP approach.

For the time series $X=\left\{x_{1},x_{2},\ldots ,x_{i},\ldots ,x_{n}\right\}$, X can be transformed into a point in the polar space P(r(i),ϕ(i),φ(i)), as presented in Figure 1, where r(i) represents the polar coordinate radius, and ϕ(i) and φ(i) stand for the angles at which the polar coordinate rotates counterclockwise and clockwise along with the initial line, respectively.

By varying the initial line’s rotation angle, a set of signals $\left(x_{i},x_{\left(i+L\right)}\right)$ can form a mirror symmetric point image in polar coordinates. The calculation formula is as the following:

(1) $$r\left(i\right)=\frac{x_{i}-x_{min}}{xmin_{max}}$$

(2) $$\phi \left(i\right)=\theta +\frac{x_{\left(i+L\right)}-x_{min}}{xmin_{max}g}$$

(3) $$\varphi \left(i\right)=\theta -\frac{x_{\left(i+L\right)}-x_{min}}{xmin_{max}g}$$

where $x_{max}$ and $x_{min}$ are the maximum and minimum values of the sampled data, respectively, L is the time interval (usually between 1–10), θ is the initial line rotation angle (values for 360m/n, m=1,⋯,n, where n represents the number of mirror symmetry planes, usually set as 6), and g is the angle magnification factor (usually set as less than θ). The differences between SDP images with various vibration forms are generally described by the arm thickness, shape features, geometric center, curvature, point concentration area, and other elements in the images.

### 2.2. Deep Residual Network (DRN)

**Figure 2:**
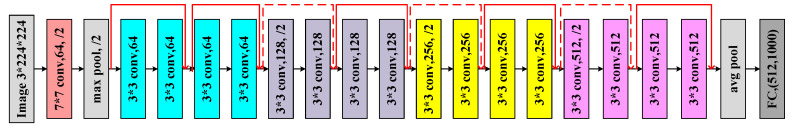
The ResNet-18 framework.

CNN is a type of feedforward neural network that contains convolutional calculation. Its nonlinear layer comprises multiple convolutional and activation layers, which can better fit very complex nonlinear functions. For noise-laden signals, such as bearing vibration signals, increasing the number of network layers allows the CNN to learn a richer set of fault features. However, deeper CNNs are more challenging to train and suffer from gradient disappearance/explosion and performance degradation. He et al. [31] presented a deep residual network (DRN) to address the mentioned issues. DRN is proposed based on CNN, as presented in Figure 2, an 18-layer residual network. The DRN mainly includes multiple residual blocks, each having multiple convolutional layers with a similar number of output channels, followed by the BN and activation layers.

**Figure 3:**
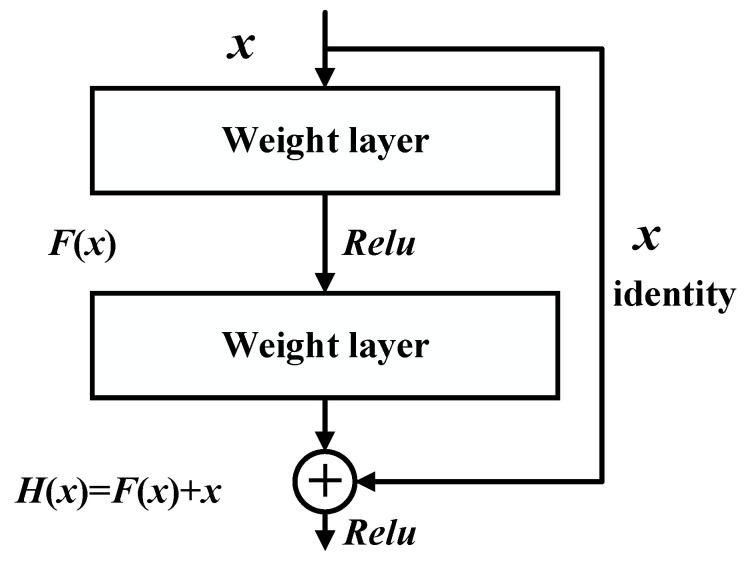
Residual block structure.

The residual block structure calculates the residuals through identity mapping, which can directly transfer part of the upper information to the last layers for fusion. The typical residual block framework is presented in Figure 3, where $F=W_{2}\sigma \left(W_{1}x\right),$ and σ, represents the Relu activation function, while the bias term is eliminated for continence. The residual block structure is conducive to the backpropagation of gradients, thus effectively updating the weights and biases and ensuring that the model can learn features as it increases in depth. The degradation problem of ordinary networks generated by an excessive number of layers is solved, avoiding the model’s precision degradation.

The convolution layer is employed for feature extraction, where a convolution kernel convolves the feature vector output from the previous layer to learn a comprehensive feature representation. The mathematical model is described as the following:

(4) $$x_{j}^{l}=f\left(\underset{i\in M_{j}}{\sum }x_{i}^{l-1}\times k_{ij}^{l}+b_{j}^{l}\right)$$

where $M_{j}$ represents the input feature vector, l is the number of layers in the network, k represents the convolution kernel, b indicates the network bias, and $x_{j}^{l}$ and $x_{i}^{l-1}$ represent the l  layer output and input, respectively. The convolutional layer has the advantages of sparse connection and weight sharing.

Batch Normalization (BN) is a method of normalizing each layer of input to be consistent in mean and variance. BN layers can accelerate the training and convergence of the network while preventing gradient explosion/disappearance and overfitting. The activation layer adopts a rectified linear unit (ReLU) activation function, expressed as follows.

(5) $$x_{i}^{l+1}\left(j\right)=max\{0,y_{i}^{l+1}\left(j\right)\}$$

where $y_{i}^{l+1}\left(j\right)$ describes the convolution operation’s output value and $x_{i}^{l+1}\left(j\right)$ represents the activation value of $y_{i}^{l+1}\left(j\right)$. The ReLU activation function makes the output of a portion of neurons zero, which can enhance the network’s sparsity, alleviate overfitting, and speed up learning.

### 2.3. Attention Mechanism

When using SDP images as input to a neural network, there are non-featured parts in the images. More importantly, to improve the model’s fault feature extraction capability and fault identification accuracy, an attention mechanism is presented in this paper to enhance the model’s diagnostic effectiveness. The attention mechanism allocates weights to different image regions, allowing the model to locate and focus on the regions with more prominent fault features and suppress useless information. The current work employs the Convolutional Block Attention Module (CBAM) as the attention mechanism [32].

**Figure 4:**
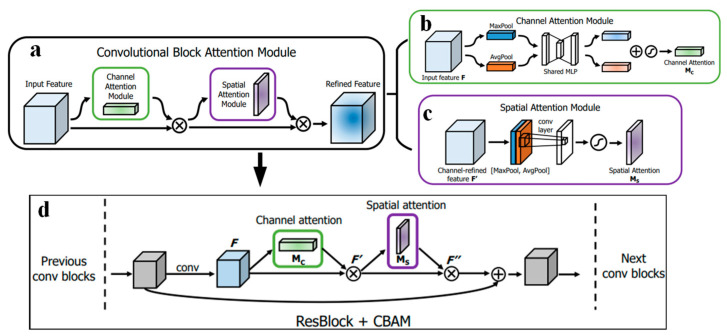
Outline of CBAM: (**a**) overall structure of CBAM, (**b**) channel attention unit, (**c**) spatial attention unit, (**d**) integrated application mode of CBAM.

CBAM is an attention unit for a feedforward convolutional neural network and its structure is presented in Figure 4a. For the input feature map, CBAM inferred the attention mapping sequentially through two independent sub-attention units (channel and spatial attention). The weights obtained using the attention mechanism are then multiplied through the input feature map for adaptive feature optimization. Given a feature map as input, CBAM consecutively deduces a 1D channel attentional map and a 2D spatial attentional map. This operation is expressed as:

(6) $$F^{\prime }=M_{c}\left(F\right)⊗F$$

(7) $$F^{\prime \prime }=M_{s}\left(F^{\prime }\right)⊗F^{\prime }$$

where ⊗ describes element-wise multiplication. The channel attention value will be passed along with the spatial dimension in the multiplication process. $F^{\prime \prime }$ represents the final refined output.

The channel attention module employs the features’ inter-channel relationships for creating a channel attention map, where its structure is presented in Figure 4b. The channel attention unit combines the feature map’s spatial information using average and maximum pooling operations, generating two spaces: $F_{avg}^{c}$ and $F_{max}^{c}$, which describe the average and maximum pooled feature, respectively. Then, they feed forward into a shared multilayer perceptron (MLP) network for generating channel attention maps $M_{c}\in {\mathbb{R}}^{C\times 1\times 1}$. In order to decrease the number of parameters, the MLP hidden layer’s activation scale is set to ${\mathbb{R}}^{C/r\times 1\times 1}$, where r  represents the reduction ratio. The output is then summed, and the feature vectors are output through a sigmoid function. Its channel attention is given using:

(8) $$\begin{array}{l} M_{c}\left(F\right)=\sigma \left(MLP\left(AvgPool\left(F\right)\right)+MLP\left(MaxPool\left(F\right)\right)\right)\\ =\sigma (W_{1}\left(W_{0}\left(F_{avg}^{c}\right)\right)+W_{1}(W_{0}(F_{max}^{c}))) \end{array}$$

where σ represents the sigmoid function, the MLP weights $W_{0}\in {\mathbb{R}}^{C/r\times C}$ and $W_{1}\in {\mathbb{R}}^{C\times C/r}$ are shared for the input, and the ReLU activation function is followed by $W_{0}$. AvgPool(⋅) and MaxPool(⋅) represent average pooling and maximum pooling operations, respectively.

The spatial attention unit employs the spatial relationships between features for generating a spatial attention map, which is complementary to channel attention. Its structure is presented in Figure 4c. The spatial attention unit performs maximum pooling and average pooling, operations that aggregate the feature mapping’s channel information, generating two 2D graphs: $F_{avg}^{s}\in {\mathbb{R}}^{1\times H\times W}$ and $F_{max}^{s^{1\times H\times W}}$, representing the channel’s average and maximum pooling features, respectively. Then, a standard convolutional layer is utilized to connect and convolve for creating a 2D spatial attention map, where the spatial attention can be determined as the following:

(9) $$\begin{array}{l} M_{s}\left(F\right)=\sigma \left(f^{7\times 7}\left(\left[AvgPool\left(F\right);MaxPool\left(F\right)\right]\right)\right)\\ =\sigma (f^{7\times 7}([F_{avg}^{s};F_{max}^{s}])) \end{array}$$

where σ  represents the sigmoid function and $f^{7\times 7}\;$ represents the convolution operation of the filter.

The CBAM is connected in series to the spatial attention unit after the channel attention unit, providing a dual channel and spatial attention mechanism, which is better than employing channel attention separately. CBAM is a lightweight, universal unit that can be conveniently incorporated into any CNN architecture and applied to the convolutional output of each block. The integrated application of CBAM is shown in Figure 4d.

## 3. Bearing Fault Diagnosis Model Using SDP Images and CBAM-DRN

**Figure 5:**
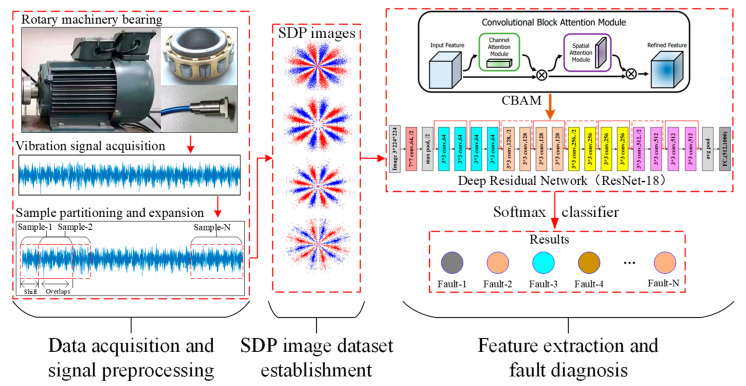
The RBFD structure using SDP and CBAM-DRN.

Based on the above SDP technique, Deep Residual Network (DRN), and CBAM attention mechanism, this section proposes an intelligent RBFD approach using SDP images and the CBAM-DRN model, where its flow chart is presented in Figure 5.

The diagnosis process of the intelligent RBFD approach using SDP and CBAM-DRN comprises three parts: (1) data acquisition and signal pre-processing; (2) SDP image dataset establishment; (3) feature extraction and fault diagnosis. The specific details are shown below.

Data acquisition and signal pre-processing: Vibration sensors are installed at suitable locations on the target-rotating machinery to collect the vibration signals during bearing operation. The bearing vibration signal is pre-processed through denoising and normalization. The sample expansion is then performed using the uniform sliding step segmentation method to increase the number of available vibration samples.

(1) SDP image dataset establishment: The SDP approach is adopted for converting bearing vibration samples into SDP images with simple structures and distinctive features and resizing them to the appropriate size. The SDP image dataset is then created and randomly categorized into training and test sets.
(2) Feature extraction and fault diagnosis: The proposed CBAM-DRN diagnosis model is established. The model is trained on the training set to obtain the ability to extract different fault state features of bearings, and the softmax classifier is then utilized for detecting different bearing faults. The whole network model is trained end-to-end using backpropagating SGD. Finally, the diagnostic efficiency of the presented approach is evaluated through the trained diagnostic model on the test set.

## 4. Comparison and Analysis

The current section presents the detailed results and analysis of the comprehensive experiments. The data used in the experiment and the parameter architecture of the diagnostic model are represented in detail in Section 4.1. In Section 4.2, the impact of several input images on the RBFD accuracy is analyzed and compared, followed by a comparison of the diagnostic efficiency of various rolling bearing fault diagnosis methods and an analytical evaluation of the diagnostic effectiveness of the several methods. In Section 4.3, the applicability of the method proposed in this paper is tested using other bearing fault data sets. All experiments are completed on PC, through the Windows 10 operating system, Intel(R) Xeon(R) 3204 CPU, and Nvidia GeForce GTX 3080 GPU. MATLAB 2017a was employed for vibration signal processing and SDP image construction. The network model is implemented using Python 3.6 in the Keras structure with TensorFlow as the back end.

In order to analyze the efficiency of the presented approach, classification accuracy was utilized to compare and verify the efficiency of the diagnostic models. Classification accuracy is the ratio between the number of correctly categorized samples and the whole number of samples in the test samples, expressed with the following equation.

(10) $$Accuracy=\frac{N_{CT}}{N_{AT}}\times 100\%$$

where $N_{CT}$ and $N_{AT}$ represent the number of truly categorized samples and the whole number of samples, respectively.

### 4.1. Experimental Setup

#### 4.1.1. Data Description

**Figure 6:**
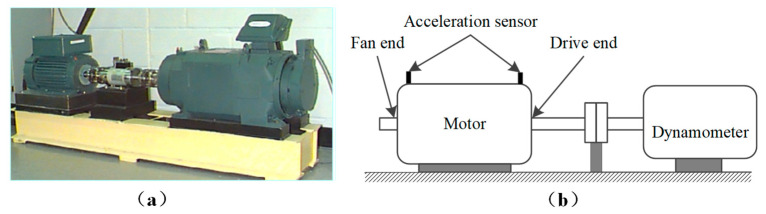
The CWRU bearing test device. (**a**) Physical experimental devices, (**b**) Structural schematic.

The experimental data of rolling bearings employed in the current paper are from the Bearing Data Center, Electrical Engineering Laboratory, Case Western Reserve University (CWRU), whose test device is presented in Figure 6. The test device comprises a 2-horsepower (hp) motor, a power test meter, a torque sensor/translator, and the corresponding electrical control unit. Three different levels of damage were pre-set on the inner ring, outer ring, and rolling element of the bearing under test, with fault diameters of 0.007″, 0.014″, and 0.021″, respectively, all with a fault depth of 0.011″. Each bearing was tested separately in experiments at loads of 0–3 hp. The experimental data contains vibration signals with various damage levels for three states of the bearing: inner ring fault (IF), outer ring fault (OF), and rolling element fault (BF). The CWRU test bearings and data descriptions are shown in Table 1.

In this paper, the rolling bearing at the drive end is employed as the research object, and the RBs’ vibration signals with various fault diameters (0.007″, 0.014″, 0.021″) are analyzed and tested at a 12 kHz sampling frequency and under various loads (0–3 hp). In order to assess the efficiency of the presented approach, an experimental dataset was created, as presented in Table 2.

The overlapping sampling technique segmented the vibration signal. Then, the four-state bearing vibration signals were changed to SDP images through the SDP approach described in Section 2. Under the four working conditions, 300 samples of normal bearing vibration signals were obtained under the four loads. Each sample contained 2048 sampling points, and 1200 samples were obtained. 3600 samples were obtained for rolling bearings with inner ring fault, outer ring fault, and rolling element fault, respectively. In order to achieve sample balance, all normal samples were employed in each experiment, and 1200 composite fault samples of the inner ring, outer ring, and rolling element with various loads and fault diameters were randomly selected. Therefore, a total of 4800 samples were adopted in each experiment in the dataset. In the experimental process, the experimental samples were categorized into the training and test sets with a ratio of 2:1. In the training process, 10% of the training samples were categorized into validation sets, adopted to verify the diagnostic model’s precision to adjust some hyperparameters in the model.

#### 4.1.2. Model Parameter Setting

This research selected an 18-layer DRN (ResNet-18) as the base model. The SDP images were resized to 224 × 224 during the model’s training step, and a CBAM-DRN model was then established for extracting the features of different fault states for RBFD. High-dimensional image features were extracted using a Conv1 convolutional layer containing 64 filters when the images were input in the diagnostic model. The filter size in Conv1 was 7 × 7, and the stride was 2. Then, the features were compressed by downsampling through a maximum pooling layer of size 3 × 3 with stride 2. Next, the deep fault features were extracted through four residual block structures (Conv2_x, Conv3_x, Conv4_x, Conv5_x) with 64, 128, 256, and 512 filters, where their size was 3 × 3. Conv3_1, Conv4_1, and Conv5_1 were downsampled in stride 2, while all other convolutional layers were downsampled in stride 1. Shortcut connections in the model perform the constant mapping. The model employs zero padding to increase dimensionality to maintain the feature map dimensions unchanged before and after convolution. Finally, all feature maps were input to the fully connected (FC) layer after an adaptive averaging pooling layer for processing and bearing state recognition using a Softmax classifier. The DRN model parameters are presented in Table 3. The batch size for model training was chosen as 64, the epoch was chosen as 500, and the learning rate was chosen as 0.001. CBAM is a lightweight, general-purpose module added to the model to improve diagnostic performance. In CBAM, the reduction ratio r in the channel attention module was 16, and the filter size in the spatial attention module was 7 × 7.

### 4.2. Results Analysis and Discussion

#### 4.2.1. Comparison of Diagnostic Performance of Different Input Images

The transformation equation of the SDP image indicates the importance of the parameters θ,L, and g. Numerous types of research have shown that the number of symmetrical figures is most suitable when θ is chosen at 60°, making the image’s symmetry and shape characteristics more prominent. Properly selected parameters g and L can enhance the graph’s resolution and intensify the differences between signals, thus better differentiating between different vibration signals. This paper employs image correlation coefficients to analyze the correlation between different images. For two images of size m×n, the correlation coefficient *R* is described as the following.

(11) $$R\left(A,B\right)=\frac{\sum_{m}\sum_{n}\left(A_{mn}-\bar{A}\right)\left(B_{mn}-\bar{B}\right)}{\sqrt{\left[\sum_{m}\sum_{n}{\left(A_{mn}-\bar{A}\right)}^{2}\right]}\left[\sum_{m}\sum_{n}{\left(B_{mn}-\bar{B}\right)}^{2}\right]}$$

where *A* and *B* are the two-dimensional gray matrices of the image. The calculated correlation coefficient *R* between the different images takes values between 0 and 1, where *R* = 0 and *R* = 1 mean that the two images are different and identical, respectively. In order to better select the optimal g and L to distinguish SDP images with different fault states, the current paper considers the sum of correlation coefficients of SDP images with four fault states as the image evaluation index, which can be expressed as:

(12) $$R_{sum}=\underset{\begin{array}{c} i\neq j\\ i<j \end{array}}{\sum }R\left(A_{i},B_{j}\right)\left(i=1,\cdots ,k-1;j=2,\cdots ,k;k=4\right)$$

**Figure 7:**
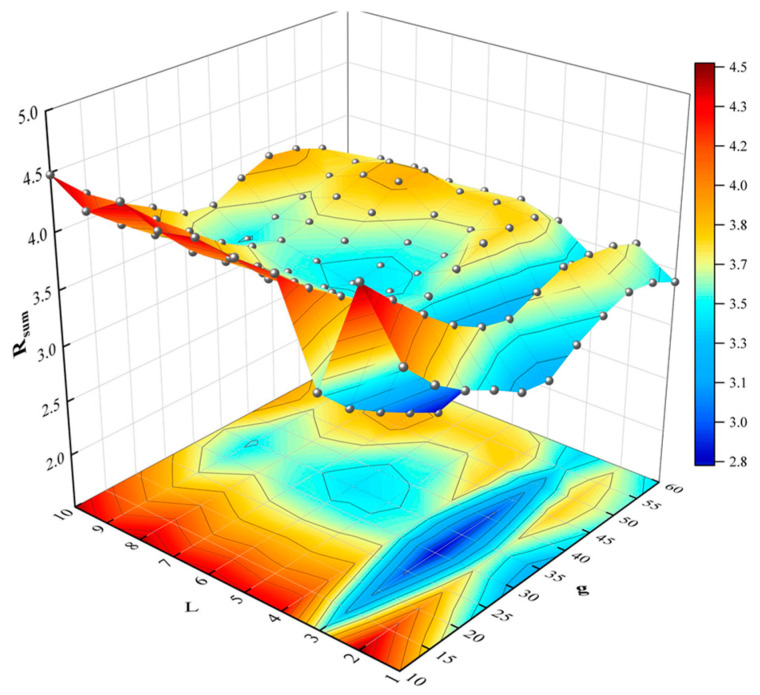
*R_sum_* for various values of g and L.

**Figure 8:**
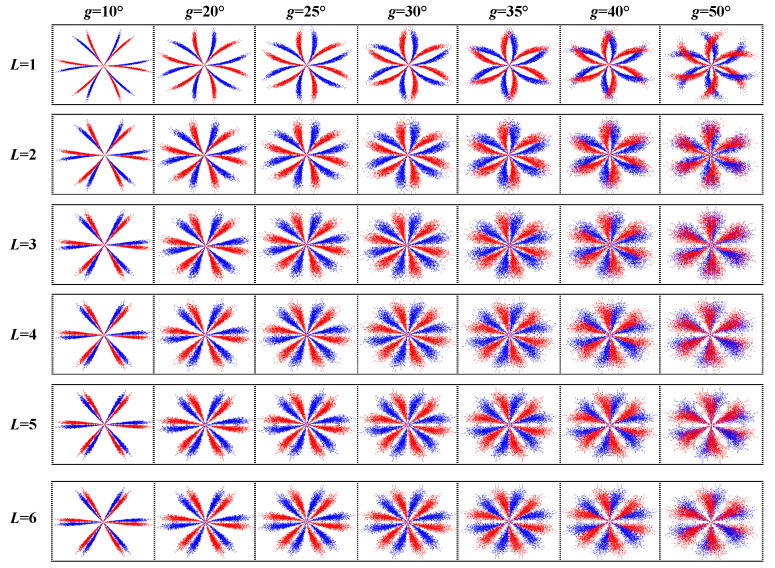
SDP images for various values of g and L.

Firstly, the bearing fault vibration signal is selected with a load of 1 hp and fault diameter of 0.007 inches, the values of *L*, *g*, and the step length are set to be 1–10, 10–60°, and 1 and 5°, respectively. Then, it is converted into SDP images, and the sum of correlation coefficients *R* of the four-fault state images is obtained. The results are presented in Table 4 and Figure 7. *R_sum_* gets a minimum value of 2.7851 when L = 3 and g = 30°. This indicates that the correlation between the different fault images is minimal, and the best identifiability is achieved when the current parameter is selected. In order to visualize the impact of parameter selection on the SDP image, some of the images are shown in Figure 8 while combining g and L. It can be concluded that the highest image quality can be obtained when L = 3 and g = 30°. As L gradually increases, the arms of the SDP image become progressively fuller. As the value of g increases, the angle between the center of mass of the SDP image arm and the horizontal axis becomes progressively larger. Therefore, the SDP parameters were chosen as θ=60°, L  = 3, and g = 30°, respectively.

**Figure 9:**
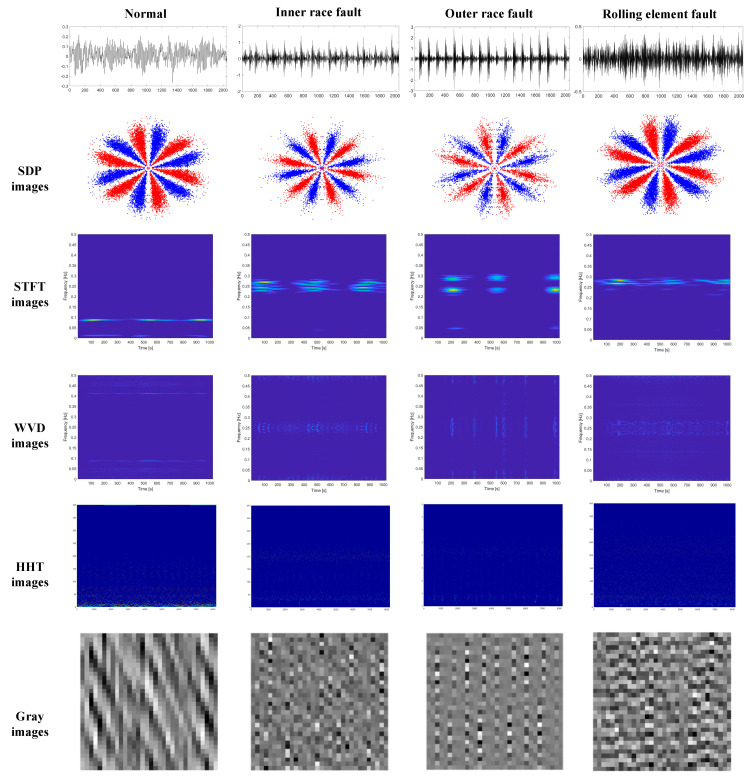
Five types of input images.

**Figure 10:**
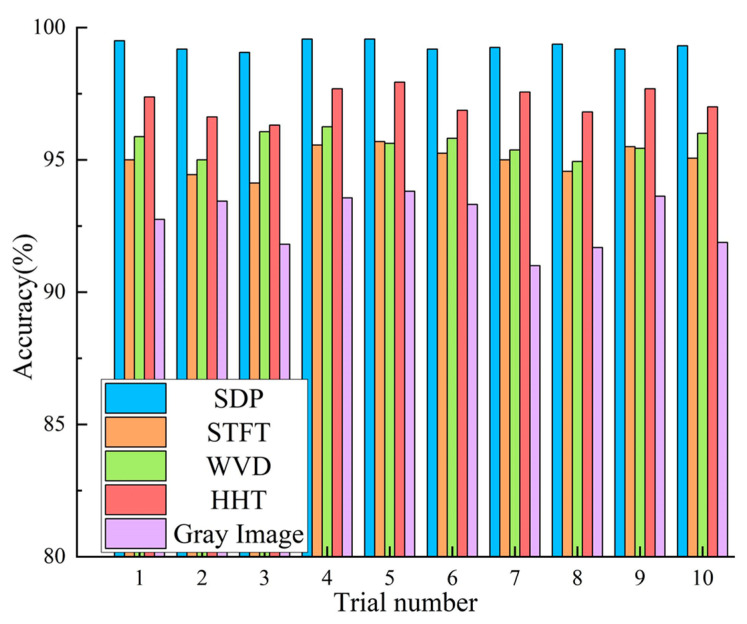
Diagnosis results of the ten experiments with various approaches.

According to the above analysis, the optimal parameters are selected to generate SDP images for RBFD. In order to clarify the benefits of the SDP images used in the current paper for RBFD tasks, rolling bearing vibration signals were converted into SDP images, Short Time Fourier Transform (STFT) images, Wigner-Ville Distribution (WVD) images, Hilbert-Huang Transform (HHT) images, and greyscale images of vibration signals [33], respectively, as input into CBAM-DRN for fault diagnosis research. Under 1 hp load, the different types of input images for the early fault of an RB (i.e., fault diameter of 0.007″ for inner ring, outer ring, and rolling element faults) are shown in Figure 9 as examples. Each experiment was performed ten times to eliminate the effect of accidental errors. The performance was assessed using the average accuracy of the ten results. Figure 10 shows the detailed diagnostic accuracies for the ten experiments. The mean accuracies and standard deviations are shown in Table 5.

The experimental results reflect that the highest detection precision is achieved using the same diagnostic model with SDP images as input. Diagnostic accuracy is around 95% for both STFT and WVD images as input. The diagnostic accuracy of HHT images as input is lower than SDP images, but higher than STFT images and WVD images, while accuracy is lower for greyscale images of vibration signals. The results indicate the excellent fault diagnosis capability of the presented approach. Since the SDP images are obtained by transforming the vibration signal into a coordinate system, the vibration signal’s fault features are not lost, and the different bearing faults can be characterized very well. In contrast, STFT and WVD are both time-frequency images. STFT truncates the vibration signal by adding a window function, while its selection influences the time-frequency image. WVD is a nonlinear time-frequency analysis method, which generates cross-talk terms when dealing with complex non-smooth signals and cannot accurately reflect the signal’s time and frequency information. Therefore, both the STFT and the WVD images are missing some information about the fault characteristics of the bearing, degrading the detection precision. In contrast to STFT images and WVD images, HHT images are obtained using the Hilbert Transform (HT) method after obtaining a series of Intrinsic Mode Functions (IMF) of the vibration signal through Empirical Mode Decomposition (EMD), since the HHT method is not limited by Heisenberg’s inaccuracy principle. Furthermore, EMD can be adaptively time-frequency localised and can effectively extract information about the features of the original signal to reflect local features. Therefore, the diagnostic accuracy of the HHT image as input is higher than that of the STFT image and the WVD image. However, EMD decomposition has the problems of mode aliasing and end effect, so the fault feature information in the HHT image will be affected, making its diagnosis accuracy slightly lower than that of SDP image.

Compared to the above methods, the vibration signal greyscale image is obtained by converting the signal’s amplitude into the corresponding greyscale value, which contains insufficient information about the bearing fault characteristics. Thus, the diagnostic accuracy is low when employing the greyscale image as input.

It is worth noting that the average diagnostic precision of the above methods exceeds 90%, which indicates that the presented CBAM-DRN is an efficient approach with good generalization and robustness. It also shows that the vibration signals of rolling bearings can be transformed into images for fault diagnosis.

#### 4.2.2. Performance Comparison of Various Fault Diagnosis Approaches

**Figure 11:**
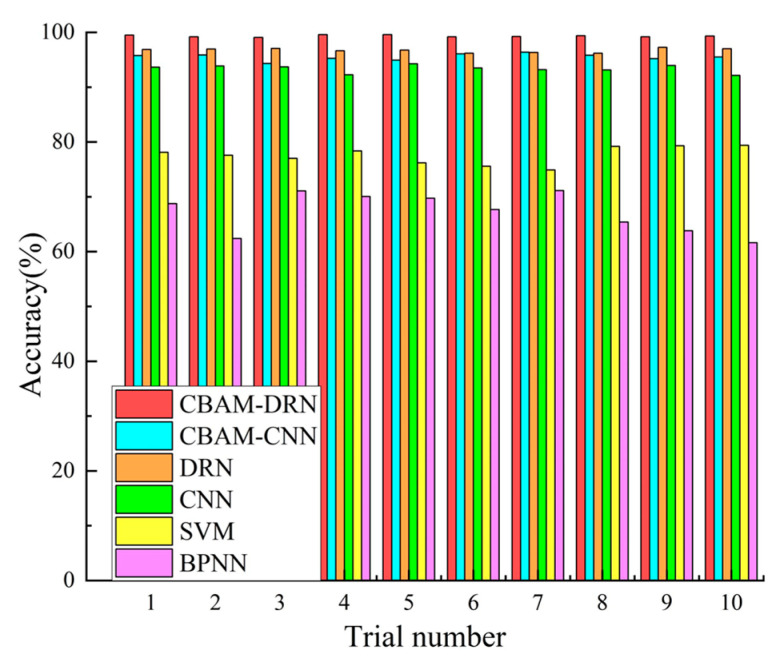
Diagnosis results of ten experiments with various fault diagnosis methods.

In order to clarify the efficiency of the presented approach, its diagnostic performance has been compared with traditional approaches using a similar data set. Among them, the CNN diagnostic model consists of five alternately connected convolutional and pooling layers, an FC layer, and a softmax classifier. The convolutional kernel size is 3 × 3, the number of convolutional kernels is 16, 32, 32, 64, and 128, the pooling layer employs a maximum pooling of 2 × 2, and the number of nodes in the FC layer is 1024. The SVM diagnostic model adopts a Gaussian radial basis function (RBF) as the kernel function. The BPNN diagnostic model is structured as 512-256-128-64-4, and the activation function is ReLU. The inputs to the SVM and BPNN are the texture feature parameters of the SDP images. All experiments were performed ten times to eliminate the effect of accidental errors. The mean accuracy of the results of ten experiments was utilized for evaluating the diagnostic efficiencies of all approaches. Figure 11 shows the detailed diagnostic accuracies for the ten experiments. Mean accuracies and standard deviations are shown in Table 6.

The experimental results show that the highest mean diagnostic precision among all experimental methods is the proposed method A (99.32%), followed by method C (96.72%), method B (95.51%), method D (93.36%), method E (77.56%), and method F (67.16%). The mean precision of the presented approach exceeds the results of several other approaches, indicating its good fault diagnosis performance. The experimental results indicate the excellent stability of the presented diagnostic approach based on SDP images and CBAM-DRN, as shown by the slight standard deviation of the diagnostic accuracy attained from various experiments. Also, it can be found from the experimental results that the diagnostic performance of the methods using the DRN model (A,C) is superior to those using the CNN model (B,D), indicating the superiority of the DRN model to the CNN model in the RBFD task. This is because DRNs have more intermediate layers than CNN models, which allows them to extract more in-depth fault features. Moreover, the DRN employs residual connectivity to allow the model to learn features even when increasing depth, which solves the performance degradation problem arising from ordinary CNNs when the number of layers increases. As presented in Table 6, methods A, B, C, and D all have good diagnostic accuracies. However, their comparison indicates that method A has a higher diagnostic precision than method C and method B has a higher diagnostic precision than method D. This is because methods A and B both add the CBAM attention mechanism, which gives different weights to different regions of the feature image, allowing the diagnostic model to locate and focus more on the image parts with more prominent fault features, thus effectively improving the diagnostic precision of the fault diagnosis model.

Notably, approaches A, B, C, and D, which employed DRN or CNN models, all had average accuracies above 90%, while methods E and F, which used SVM or BPNN models, all had accuracies below 80%. This is because both DRN and CNN are deep diagnostic models. Compared to shallow models such as SVM and BPNN, deeper models can extract more and deeper fault features, thus effectively characterizing the complex mapping relationships between bearing vibration signals and fault states. Moreover, since the data samples employed in the experiments were randomly selected from a composite fault dataset with different loads and fault diameters, the deeper model with better generalization capability for fault diagnosis can achieve higher diagnostic accuracy. Besides, compared with SDP images directly input into the deep model, artificial feature extraction and complex signal processing approaches determine the diagnostic efficiency of conventional approaches like SVM and BPNN. This experiment adopts the texture feature parameter of the SDP image as the input for the SVM and BPNN, introducing uncertainty due to human interference in the extraction process. Furthermore, texture features are manually extracted features designed for a specific diagnostic model, which is time-consuming, labor-intensive, and not universal.

**Figure 12:**
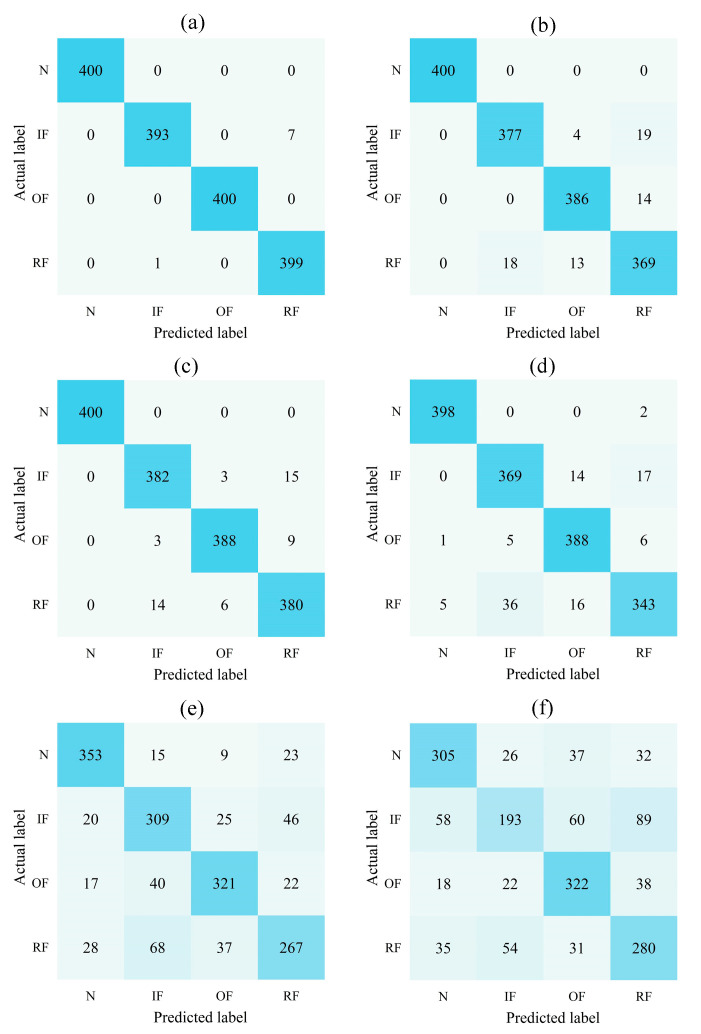
Confusion matrix of the different fault diagnosis methods. (**a**) CBAM–DRN, (**b**) CBAM–CNN, (**c**) DRN, (**d**) CNN, (**e**) SVM, (**f**) BPNN.

In order to analyze the classification of the above fault diagnosis methods in more detail, the classification results of the different diagnosis methods were counted during the first experiment to attain the confusion matrix, as shown in Figure 12. The vertical coordinate of each confusion matrix indicates the classification’s actual label, and the horizontal coordinate indicates the predicted label. The elements on the main diagonal of the confusion matrix indicate the number of samples in the current category that were correctly classified. The confusion matrix of the presented approach provides a better classification of samples for each type of health status and the highest number of correctly classified samples, indicating its high classification accuracy. This illustrates the validity and applicability of the presented diagnostic approach for distinguishing between different types of rolling bearing health states.

**Figure 13:**
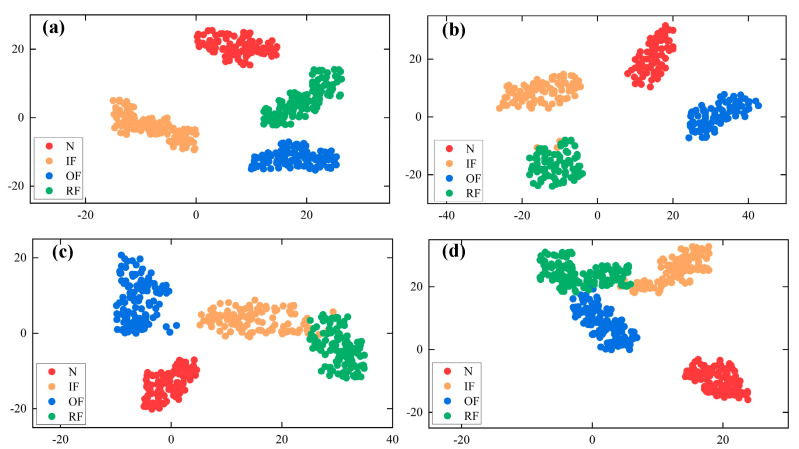
Feature classification visualization using t–SNE. (**a**) CBAM–DRN, (**b**) DRN, (**c**) CBAM–CNN, (**d**) CNN.

In order to further clarify the efficiency of the presented diagnostic approach and get a more intuitive understanding of its feature extraction and fault classification capabilities, the t-SNE technique [34] was utilized to downscale and visualize the fault features extracted for CBAM-DRN and other methods using deep models, as presented in Figure 13.

The visualization of the CBAM-DRN shows that features of the same type are aggregated with small intra-class distances, while the fault features of different states are effectively separated using the diagnosis method with large inter-class distances. In contrast, other methods confuse and misclassify the feature maps of different fault categories. It indicates that the presented approach has better fault feature extraction and classification capability under the multi-load and multi-fault diameter conditions. It is proved that different bearing faults can be well represented by transforming bearing vibration signals into SDP images without losing the critical fault feature information in vibration signals. It also shows that the SDP image has more representative and discriminative fault feature information than the manually extracted fault features.

### 4.3. Validation of Diagnostic Method Applicability

**Figure 14:**
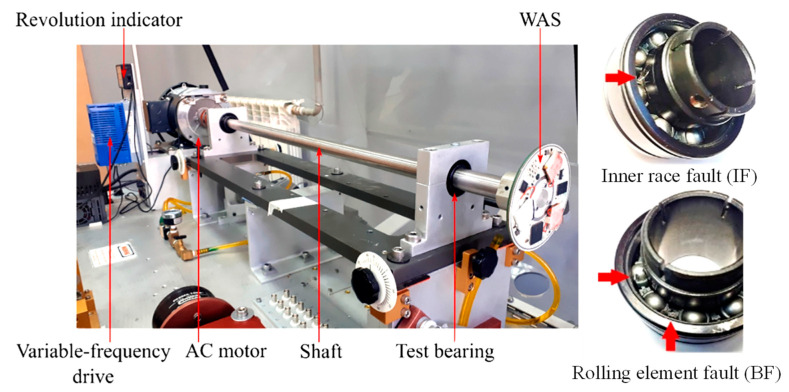
Bearing fault experiment rig and faulty bearings.

In order to verify the applicability of the proposed bearing fault diagnosis method when applied to different types of bearings, experiments were conducted using a bearing fault database from South Ural State University [35,36]. The experimental rig used to collect the bearing fault data is shown in Figure 14. The rig contains two bearing assemblies with a 1″ shaft. The shaft is supported by a bearing assembly and is rotated using an AC motor drive. The bearings were selected from the commercial ER-16 K bearing from MB Manufacturing. The normal bearing is on the left side of the test bench and the faulty test bearing is on the right side. The AC motor is fixed to the shaft using a jaw coupling adjacent to the normal bearing. A rotation indicator is used to measure the frequency of the rotating shaft. The WAS-prototype contains three one-axis MEMS-accelerometers ADXL-001 (Analog Devices) mounted on the shaft end of the test bearing for signal acquisition. Different bearing fault states are simulated by applying mechanical damage to the test bearing with a rotating tool.

**Figure 15:**
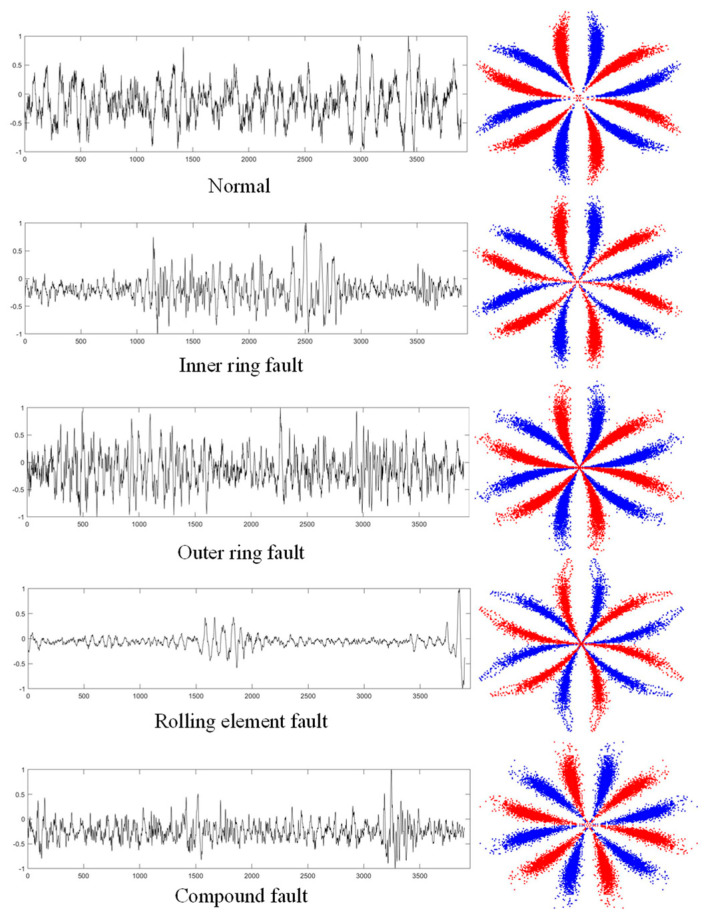
Bearing vibration signals and SDP images with different faults.

When sampling the bearing signals, the shaft of the experimental rig was rotated at 1200 rpm (20 Hz) and 1080 rpm (18 Hz), respectively, with a sampling frequency of 31.175 kHz. The database contains five types of bearing operating data. These are normal condition (N), inner ring fault (IF), outer ring fault (OF), rolling element fault (RF) and compound fault (CF) which contains all three faults at the same time. In this paper, the bearing vibration signal at 20 Hz is selected for experimental analysis. Firstly, the acquired bearing vibration signals are pre-processed, and the vibration signals are divided into samples using overlapping sampling techniques. Then the five states of the bearing vibration signals are converted to SDP images using the SDP method, as shown in Figure 15.

The bearing vibration signals of different operating conditions were processed to obtain SDP image samples. Separately, 1500 samples were randomly selected from each category to build the experimental data set as shown in Table 7. Each sample contains 3897 sampling points. The dataset contains SDP images of normal and four fault state bearings, with images sized to 224 × 224, and a total of 7500 samples. In the experiment, the training set and the test set were divided into a 2:1 ratio for the experimental samples.

**Figure 16:**
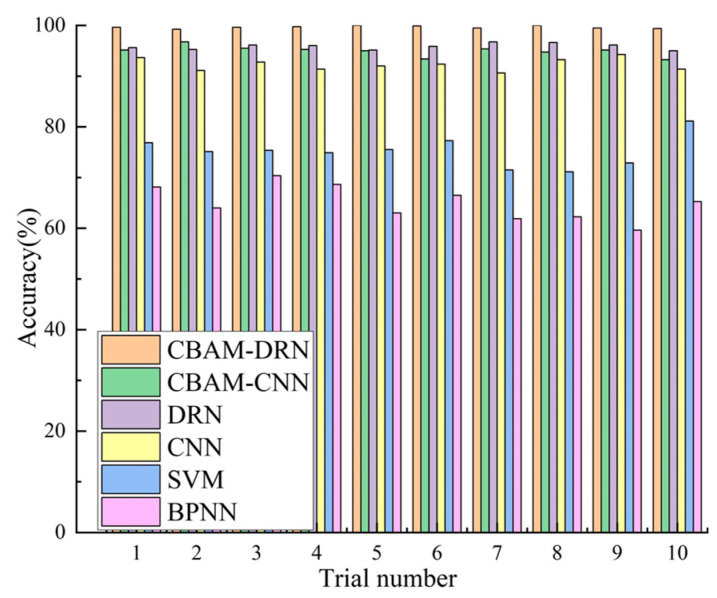
Diagnosis results of the 10 trials using different methods.

Validation of the applicability of the bearing fault diagnosis method proposed in this paper based on the above data set. The SDP image conversion parameters and the CBAM-DRN diagnostic model used in the research is set with the same parameters as in the previous section. To illustrate the effectiveness and applicability of the proposed method, the diagnostic performance of the proposed method was compared with other methods such as CNN, SVM, and BPNN based on the same dataset, and their parameter set was the same as in the previous section. In order to eliminate the effects of chance errors, 10 trials were carried out for each method. The average accuracy of the results of the 10 trials was used as an indicator of the effectiveness of each method to assess its diagnostic performance, and the experimental results are shown in Figure 16 with Table 8.

From the experimental results, it can be seen that the bearing fault diagnosis method proposed in this paper can still achieve the highest diagnostic accuracy (99.65%) compared to other diagnostic methods. The proposed method has the smallest standard deviation of diagnostic accuracy, indicating that the method has good stability. It is shown that the residual linking and attention mechanism used in the proposed method enables the diagnostic model to extract comprehensive and deep fault features from the SDP images, thus effectively improving the diagnostic accuracy.

**Figure 17:**
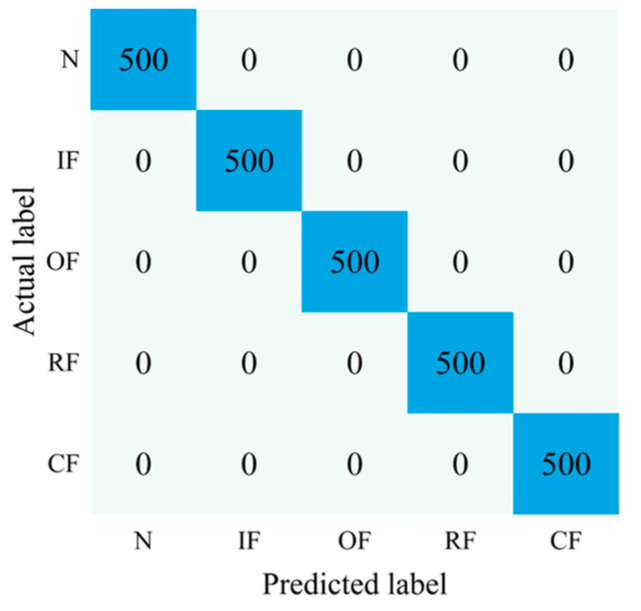
Classification confusion matrix of CBAM-DRN in the first experiment.

To better illustrate the good diagnostic accuracy and classification accuracy of the bearing fault diagnosis method proposed in this paper, we give the confusion matrix of the classification results of the proposed method in the first experiment, as shown in Figure 17. It is clear from this that the proposed method can accurately classify various types of fault samples, which indicates that the proposed diagnostic method can effectively diagnose and classify different types of rolling bearing health conditions.

All the above results illustrate that the rolling bearing fault diagnosis method based on SDP images and CBAM-DRN diagnosis model proposed in this paper can also achieve outstanding fault diagnosis accuracy on other bearing data sets and can accurately classify bearing faults in different states. It is evidenced that the fault diagnosis method proposed in this paper has good applicability and can be well applied to different categories of bearing fault diagnosis tasks.

## 5. Conclusions

For accurate RBFD in rotating machinery, the current work presents an RBFD approach using SDP images and CBAM-DRN. The method converts rolling bearing vibration signals into SDP images and employs them as input. CBAM-DRN is then employed as a diagnostic model to diagnose faults in rolling bearings.

The SDP method can quickly visualize the bearing vibration signal and convert it into an SDP image with intuitive and significant fault features. The DRN overcomes the performance degradation caused by stacking layers in a normal CNN and entirely derives the fault characteristics in the image using the deep structure. Besides, the incorporated attention mechanism can make the diagnostic model focus more on the significant components of the fault features, suppressing useless information and enhancing the performance of fault diagnosis. Finally, the effect of various input images and fault diagnosis methods on the RBFD accuracy is verified through the motor-bearing composite fault dataset. The experimental results reflect that the presented approach provides a recognition rate higher than 99% for bearing fault samples with different loads and fault diameters, as well as good generalization and robustness.

At the same time, the severe noise disturbance in the actual operating situations will be considered, and the application of the RBFD approach to the rotating equipment in the actual working conditions will be researched. This provides an excellent diagnostic capability for them even in the complex environment of industrial sites.

## Figures and Tables

**Table 1:** CWRU test device and data description.

Parameter	Bearing Position	
	Drive End	Fan End
Bearing type	6205-2RS JEM SKF	6203-2RS JEM SKF
Inside Diameter	0.9843″	0.6693″
Outside diameter	2.0472″	1.5748″
Thickness	0.5906″	0.4724″
Pitch diameter	1.537″	1.122″
Ball number	9	8
Ball diameter	0.3126″	0.2656″
Motor speed (0–3 hp)	1797/1772/1750/1730 rpm	1797/1772/1750/1730 rpm
Sampling frequency	12/48 kHz	12 kHz
Fault diameter	0/0.007″/0.014″/0.021″	0/0.007″/0.014″/0.021″

**Table 2:** Rolling Bearing fault sample dataset.

	N	IF	OF	BF
Load	0 HP/1 HP/2 HP/3 HP			
Fault diameter	0	0.007 in./0.014 in./0.021 in.		
Number of samples	1200	3600	3600	3600
Randomly selected samples	1200	1200	1200	1200
Training set	800	800	800	800
Testing set	400	400	400	400

**Table 3:** The architecture of the DRN diagnostic model.

Layer Name	Specification	Output Size
Inputs	-	224 × 224
Conv1	7 × 7, 64, s = 2	112 × 112
Max pool	3 × 3, s = 2	56 × 56
Conv2_x	$\left[\begin{array}{cc} 3\times 3, & 64\\ 3\times 3, & 64 \end{array}\right]\times 2$	56 × 56
Conv3_x	$\left[\begin{array}{cc} 3\times 3, & 128\\ 3\times 3, & 128 \end{array}\right]\times 2$	28 × 28
Conv4_x	$\left[\begin{array}{cc} 3\times 3, & 256\\ 3\times 3, & 256 \end{array}\right]\times 2$	14 × 14
Conv5_x	$\left[\begin{array}{cc} 3\times 3, & 512\\ 3\times 3, & 512 \end{array}\right]\times 2$	7 × 7
Outputs	-	4

**Table 4:** *R_sum_* for various values of g and L.

*R_sum_*		L									
		1	2	3	4	5	6	7	8	9	10
g	10	4.0028	4.5171	3.5341	4.3481	4.3549	4.4003	4.341	4.469	4.285	4.4811
	15	3.7231	4.252	3.2602	4.1074	4.0653	4.1177	4.0536	4.2099	4.0599	4.2167
	20	3.5362	4.0108	3.0841	3.9168	3.8576	3.8739	3.8531	3.994	3.856	4.027
	25	3.3971	3.7948	2.9303	3.7248	3.6974	3.6697	3.6885	3.7802	3.6601	3.8729
	30	3.2341	3.6471	2.7851	3.5613	3.492	3.4949	3.5161	3.6293	3.5084	3.707
	35	3.1911	3.5825	2.8481	3.5338	3.4521	3.4038	3.4877	3.5632	3.4645	3.6694
	40	3.3582	3.6836	2.908	3.6467	3.4855	3.4428	3.5542	3.6165	3.5497	3.8109
	45	3.4755	3.775	3.0452	3.7529	3.5234	3.5306	3.696	3.7344	3.6244	3.9127
	50	3.5534	3.7574	3.2209	3.7712	3.6399	3.6695	3.8707	3.8275	3.7168	3.8744
	55	3.5099	3.7156	3.4368	3.7473	3.7752	3.77	3.8618	3.8429	3.7418	3.778
	60	3.3923	3.62	3.3685	3.6054	3.6948	3.6718	3.6545	3.6918	3.6719	3.5684

**Table 5:** Experimental results with four different inputs.

Method	Average Accuracy (%)	Standard Deviation (%)
SDP + CBAM-DRN	99.32	0.1664
STFT + CBAM-DRN	95.02	0.4874
WVD + CBAM-DRN	95.64	0.4219
HHT + CBAM-DRN	97.19	0.5093
Gray Image + CBAM-DRN	92.69	0.9560

**Table 6:** Diagnosis results of different methods.

Methods	Model	Average Accuracy (%)	Standard Deviation (%)
A	CBAM-DRN (Proposed method)	99.32	0.1664
B	CBAM-CNN	95.51	0.5718
C	DRN	96.72	0.3593
D	CNN	93.36	0.6656
E	SVM	77.56	1.5295
F	BPNN	67.16	3.4178

**Table 7:** Bearing failure sample data set.

Fault State	Training Set	Testing Set
N	1000	500
IF	1000	500
OF	1000	500
BF	1000	500
CF	1000	500

**Table 8:** Experimental results of different fault diagnosis methods.

Model	CBAM-DRN	CBAM-CNN	DRN	CNN	SVM	BPNN
Average accuracy (%)	99.65	94.95	95.85	92.28	75.16	64.96
Standard deviation (%)	0.2423	0.9637	0.5695	1.1247	2.7934	3.2452

## Data Availability

Data used in this research were acquired from the Case Western Reserve University (CWRU) Bearing Data Center and South Ural State University (SUSU), respectively. The network connection is http://csegroups.case.edu/bearingdatacenter (accessed on 18 March 2021) and https://github.com/susu-cm/bearings-dataset (accessed on 11 September 2022) respectively.
